# Saving Women, Saving Families: An Ecological Approach to Optimizing the Health of Women Refugees with S.M.A.R.T Primary Care

**DOI:** 10.3934/publichealth.2016.2.357

**Published:** 2016-06-01

**Authors:** J. Nwando Olayiwola, Melanie Raffoul

**Affiliations:** 1 J. Nwando Olayiwola, MD, MPH, FAAFP. University of California, San Francisco, Department of Family & Community Medicine,1001 Potrero Avenue, Building 80, San Francisco, CA 94110; 2 Melanie Raffoul, MD. New York University, Department of Emergency Medicine

**Keywords:** refugee, women, primary care

## Abstract

More than 43 million people worldwide have been forcibly displaced from their homes as a result of conflict and persecution, over 50% of whom are women and 41% are children. The United Nations estimates that two-thirds of the world's refugees have been in exile for over 5 years, and more than half are in urban environments, as opposed to camps. Therefore, long-term strategies for healthcare in receiving countries are needed. The unique challenges facing refugee women as they seek safe and stable living situations are compelling. A system that optimizes the health of women refugees has significant implications for the rest of the family.

## 1. Introduction

*“When you invest in women and girls, you invest in everyone.”* — Melinda Gates.

The world is facing an unprecedented refugee crisis due to destabilized home countries in the Middle East and North Africa. More than 43 million people worldwide have been forcibly displaced from their homes as a result of conflict and persecution, which the United Nations states is the highest number since the mid-1990s. Over 15 million of these people are uprooted refugees who fled their homelands. Over 50% of refugees worldwide are women and 41% are children [1].

The UN estimates that two-thirds of the world's refugees have been in exile for over 5 years, and more than half are in urban environments, as opposed to camps [1]. This presents a need for long-term strategies in receiving countries, as refugee status is likely to be increasingly prolonged. However, many host countries or resettlement organizations have focused on refugee health through the traditional medical model, which involves short-term access to care or screening patients as they present for care, cataloging their medical history and potential risk of contracting or transmitting infections to others, administering vaccines and getting laboratory studies, screening for trauma, or providing brief wellness and chronic disease care [2]. While these comprehensive short-term medical programs are essential, many are not formally integrated with the social service sector. In parallel streams, the integration of refugees into larger societal constructs is often the work of social service entities, foundations or non-governmental organizations (NGOs). Nations contending with a refugee or migrant influx will make decisions that will impact displaced people and the existing infrastructures of health, education, workforce, environment, and housing. Preemptive planning, as opposed to post influx reaction, is necessary to guide outcomes, rather than simply dealing with impact.

Current estimates of the numbers of women and their children refugees make a focus on the health of women, as a precursor for healthy families, imperative. The unique challenges facing refugee women as they seek safe and stable living situations are compelling. For women, rates of trauma, joblessness, domestic violence, sexual exploitation and violence, fear, isolation, poor literacy, inadequate childcare, and insufficient primary health care are high [3]. Refugee women and girls are less likely than refugee men and boys to have access to even the most fundamental of their rights such as the right to food, health care, shelter, nationality, and documentation [3]. Additionally, they are highly prone to discrimination and more likely to have been caught up in conflict in their country of origin. Women and girls are more often subject to sexual violence, including domestic violence and human trafficking, as well as a variety of harmful traditional practices, including female genital mutilation, forced or early marriage, “corrective rape”, and “honor crimes”. Further abuse is common when in displacement. This is all added to the baseline level of discrimination of which women are at higher risk, including discrimination found in legal systems, in access to work permits, at health centers, in schools and in leadership positions.

All women, whether single or married, young or elderly, abled or disabled, heterosexual or lesbian/gay/bisexual/transgender (LGBT), mothers or childless, are at risk for significant physical, mental and social atrocities during this process of flight, displacement and resettlement. For those with children, the risks are often compounded, and children are often highly vulnerable to abuse, exploitation, neglect or unsafe conditions, and are more likely to bear the consequences of adverse childhood experiences into their adult lives [4].

Therefore, as women seek asylum and resettlement in host countries, it is essential that there is a systems approach their health care needs that is more comprehensive than a traditional medical model can offer. In this paper, we propose tackling the health of women refugees through an ecological model, in which the primary health care system can serve as a gate of entry to the myriad of other services and needs of these women and their families, and apply S.M.A.R.T and strategic thinking to service provision. Stabilizing and strengthening women presents an optimal opportunity to improving the stability and success of their households. We acknowledge that comprehensive intervention assumes advanced resources. Unfortunately, many accepting nations, particularly developing ones, may not always have the strong social infrastructures needed to accomplish this [5]. Our paper, therefore, focuses on refugee health and resettlement in more developed or higher income nations.

## 2. An Ecological Framework and Refugee Integration

**Figure 1.**
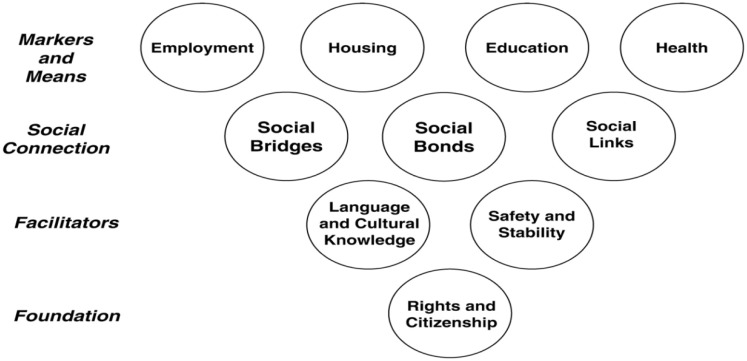
A Conceptual Framework Defining Core Domains of Integration. Source: Ager and Strang. Reprinted with permission [7],[9].

There is a lack of ample data describing the systematic features of successful integration of refugees into host countries according to the United Nations High Commission on Refugees (UNHCR) [6]. However, commonly used frameworks for successful integration, generally include four major themes and were well articulated by Ager and Strang in 2008: 1) achievement across multiple sectors such as housing, employment, education and health; 2) Citizenship and rights processes and practice; 3) Social connections within and between community groups; and 4) Structural barriers such as language, culture and understanding of the local environment (Figure 1) [7],[8].

Several protective factors and “cross-cutting” areas for refugee integration have been identified. Protective factors for refugees include maintenance of family cohesion, stable living environment post resettlement, social support, community integration, and economic opportunity with maintenance of socioeconomic status [10]. Conversely, multiple barriers to health and healthcare can be found, among them mistrust and perceived discrimination, limited health services, unaffordability of health services, inadequate shelter, food insecurity, unemployment, and inadequate community support [11]. Success in cross cutting areas affects advancement in other integration areas [6]. Cross-cutting areas include family separation or unification, appropriate documentation, language, health, time spent in the asylum process, and transitioning out of reception housing. Prior assessments of integration have looked at education, employment, housing, and family reunification. Others have described legal, economic, and social and cultural process to integration [6].

A shared agenda and collaboration between stakeholder representatives from primary care, mental health, social work, education, employment, housing, and local and state governments allows for comprehensive interventions on refugee health [12]. Similar collaborations have been called for when tackling global development objectives, and in the global development arena, the empowerment of women has emerged as a key element to goal attainment [13],[14]. When women are empowered, there are numerous beneficial by-products, including the likelihood that their children will thrive and their families will more readily access healthcare and other opportunities. Additionally, empowered women are less likely to remain in households with domestic violence or other forms of sexual violence and exploitation [15]. The empowerment of women and mothers has been a strong predictor of health promotion, positive health outcomes and social action [16],[17]. Women have historically required a multiplicity of interventions and special attention to optimize their transition and resettlement as refugees, because of complexities at the intersection of their gender, socioeconomic status and pre-migration realities [18].

**Figure 2.**
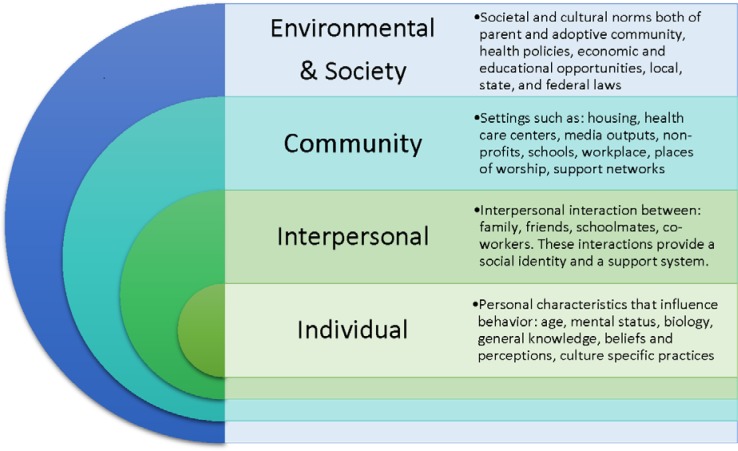
An Ecological Framework for Optimizing the Health of Women Refugees.

For most nations, the primary health care system is the entry into the larger healthcare system and serves as an important foundation for health equity, coordination of comprehensive responses at multiple levels, and confluence of multiple sectors and influences on people's lives [19]–[21]. Therefore, primary health care, as Starfield and colleagues described [19], is often the first point of healthcare contact newcomers make in a host country, and facilitates entry into the rest of the system. Seizing the opportunity of primary health care to influence the health, well-being, integration and success of refugee women may be just what systems contending with large influxes of refugees need. However, limiting this to a traditional medical model of providing screening and brief treatment will not be sufficient, as many women refugees in host countries experience unmet health needs [22]. While the primary care setting may not be appropriate for foundational elements of the Ager integration framework such as citizenship, many linkages and collaborations facilitated by the primary health care setting can ultimately contribute to a successful pathway for women and their families. In this paper, we offer thinking through an ecological model (Figure 2), which outlines some considerations for successful integration of women refugees into their host countries with primary health care settings serving as the gateway. Host countries may anticipate special needs of women during periods of high evacuation and flight, and resource planning for the primary care setting should follow accordingly.

## 3. Components of the Ecological Model

### 3.1. Individual lifestyle factors

Individual dimensions of the model focus on personal characteristics that influence behavior such as age, mental status, biology, general knowledge, beliefs and perceptions, culture specific practices.

Traditional medical models of health care focus on the individual in terms of their genetics (personal, familial, cultural), biological factors and predispositions, habits and risk factors. Screening, diagnosis and treatment plans are invariably linked to these factors. An ecological approach to addressing individual factors for refugee women requires that refugees are viewed as individuals, and not as a homogenous group [6]. The primary care team must seek to understand both personal decision-making capacities, circumstances surrounding flight from the country of origin, and socio-cultural nuances of communication. Personal choice is often dictated by both natural and environmental/situational phenomena. For many refugees, both the influences of their parent culture and their experience in transition will shape their personal choices and behaviors. An understanding of their culture and beliefs will allow for competent and sensitive communication and the identification of potential barriers to care.

Women are often in charge of meal preparation, child rearing, household management, and accessing healthcare. Lifestyle choices as they relate to diet, disciplining of children, and appropriate use of healthcare can have a significant impact on health and wellbeing. Screening for food security and resources is essential for refugee women. Many refugees suffer nutritional deficiencies or excesses when faced with Western diets and are often victims of food insecurity [23]. Counseling women on the practicalities of shopping for food and the various resources in the community, in addition to making good dietary choices, can positively impact women and their families. Consideration of balance between healthy foods and ethnic cuisine is important to ensure women and their families maintain affinity and fidelity to the foods that they know. Community specific resources, such as farmers markets or food pantries can help families access healthy foods for reasonable costs.

Discussing alcohol and drug use and addiction morbidity can affect choices that women make during transition, which is a high-risk period for substance abuse. Flight, transition and resettlement of refugees are frequently accompanied by psychosocial distress, challenges of acculturation and mental health disorders [24],[25]. Screening refugee women and empowering women to assess for substance abuse in their children, especially adolescents, who are at high risk, is an important contribution of primary care to their overall health.

Additionally, comprehensive approaches to refugee women's health should include culturally competent and language concordant obstetric and gynecological care, pre-conception counseling and care, discussion on child-rearing beliefs and practice, sexual health history and experiences, family planning values and goals, physical activity and nutrition education [26], cancer and other screenings and vaccinations [18].

### 3.2. Interpersonal Networks

Interpersonal dimensions of the model include interpersonal interactions between tighter groups such as: families, friends, schoolmates, neighbors, and co-workers. These interactions provide both a social identity and a support system.

Lifestyle choices refer to the workings of the household and family unit, while social and community networks involve the interaction between a household and its neighbors. In a traditional medical model of primary care, interpersonal networks are less relevant to service delivery than individual characteristics. However, applying an ecological lens to the health of women refugees undeniably requires an understanding of the interpersonal skills, resources and networks the women are engaged in. Though newcomers have different experiences that lead them to their new countries, they share challenges in rebuilding social networks and in the experience of social exclusion based on race/ethnicity, religion, language, immigrant status and resource limitations [27].

Primary care settings providing service to refugee women and their families should screen for and understand at least two categories of “community”: the parent community and the adoptive (host) community. Maintaining connections with families left behind is as important as creating new connections. In some cases, loss of connection to the parent country and social supports is likened to “death” [28],[29]. Primary care teams and their partners can connect new refugee women with others who have successfully navigated or are also currently negotiating life in their new environment, while also balancing native identity and relationships. Cohesive groups of women navigating this process together can lead to collaborations, shared stories, encouragement, and community. In addition, refugee women who have transitioned and have become a part of a larger community can be in a position to serve that community as a liaison between newer refugees and community representatives, identifying areas or persons of need, and mentoring new women.

Primary care settings can also serve as community hubs for meetings and connecting refugees with other women in their new country, and provide pathways for active citizenship and civic engagement. “Active citizenship” has been described as a marker of successful integration. Voting, interaction with government organizations, and taking an interest in local affairs are all examples of active citizenship. Lack of language fluency, caution regarding cultural norms, and isolation can impede active citizenship. Enabling women to achieve active citizenship in their communities is a powerful marker for later success, and active engagement of refugee women has great potential for host nations [7],[14],[30].

### 3.3. Community

Community dimensions of the model include: housing, health care centers, media outputs, non-profits, schools, workplace, places of worship, and a variety of other external support networks.

The breadth of potential community involvement spans multiple arenas, including housing, sanitation, employment, worship, healthcare services, schools, and work environments. Every refugee becomes part of a larger community and must acquire or enhance certain skills to be successful in it.

Language plays a significant role in the ability to integrate into a new community, and is also the strongest barrier to integration. Without a working proficiency in the language of the host country, a refugee cannot effectively maneuver employment, education, health, and other social interactions that are core to their success [6]. Screening for language proficiency and skills in the host country is an essential part of an integrated model of refugee integration, and structuring programs that address deficiencies and gaps, tailored to educational level, is important. Primary care settings can incorporate language proficiency screening into refugee assessments and design language programs that support the second language. Additionally, they can provide language interpretation services at the point of care and in between care transactions [31].

Language programs also provide a platform for community building and shared learning activities. Collaborative programs between medical, social and educational services may optimize such programs. For example, health care centers offer a sensible setting for language courses for women refugees, and may be coupled with health education, community cooking, computer and technical literacy, wellness courses, and other activities. Alternatively, places of worship or educational centers can also incorporate health related training and education, language instruction and more, during or after worship based programs. Healthcare and community partnerships may facilitate these types of arrangements.

To the extent that it is possible, host countries can offer or extend services to refugees that assist in community building. For example, connecting women through social networks to share their own stories and challenges, and to assist with way finding and community engagement has ripple effects for families. Such communities may help women retain elements of their culture, provide guidance on educational and vocational opportunities as well as explore economic pathways.

Employment is a key concern among refugees. Economic productivity leads to dignity, self-reliance, and upward mobility. Stable employment positively affects both refugees and receiving societies [6]. The attainment of employment can be difficult and guided efforts are most efficient at matching refugees to vocational and skill based work sectors. For some women, this may be the first time they venture into the workplace or have the predominant responsibility for maintaining the financial health of their family. For others, resettling may be coupled with a significant decline in class or societal position, and many refugees are devastated by loss of meaningful employment and previous occupational status when starting over in their new countries [32].

Resources in the healthcare setting to encourage and guide women through the process of employment is important, with special consideration given to the ancillary needs women in the workforce have, namely childcare, nursing needs, financial management and transportation assistance. For refugee women, primary care teams can also support those who have found employment by inquiring about factors that they are often vulnerable to, such as excessive workloads, low wages, devaluation, discrimination and abuse [33], and providing support to obtain recourse when indicated.

### 3.4. Environment and society

Finally, environmental and larger societal dimensions of the model include health policies and other policies, local, state and federal laws, and societal and cultural norms of the parent and adoptive community.

While the healthcare delivery system has an important role in organizing, developing or connecting services that optimize comprehensive care of women in refugee status, health and other policies are important to sustain and spread best practices. Many women refugees are unaware or unable to understand policies and laws of their new environments, including which benefits they may be eligible for or protective laws that may apply to them [34].

Providing refugee women with orientation on Federal, state and local laws, such as those on gender violence, torture survivorship and human trafficking, may be directly beneficial to them, as much of this is underreported in refugee communities [35]. Primary care settings can host or connect women to programs that teach self-defense and other important skills for their protection, such as a successful UNHCR program in New Delhi, India [36]. In the United States, laws regarding cultural and linguistic standards (CLAS) and services may be encouraging to newcomers [37]. Additionally, expansion of CLAS standards may be a useful lever for strengthening services offered to women refugees and their families [38] while also making these women feel more at home. Women refugees may also be eligible for direct federal or state resources such as interim cash, housing assistance, childcare benefits and more. Additionally, certain local laws regarding safety and security may not be clear to new women, so ensuring their exposure to these is also important.

Similarly, education on cultural norms, values and laws should also be extended to men who perpetrate violence and other crimes against women. An interesting program for training male Sudanese refugees in Norway on appropriate treatment of women may be a model [39].

**Figure 3.**
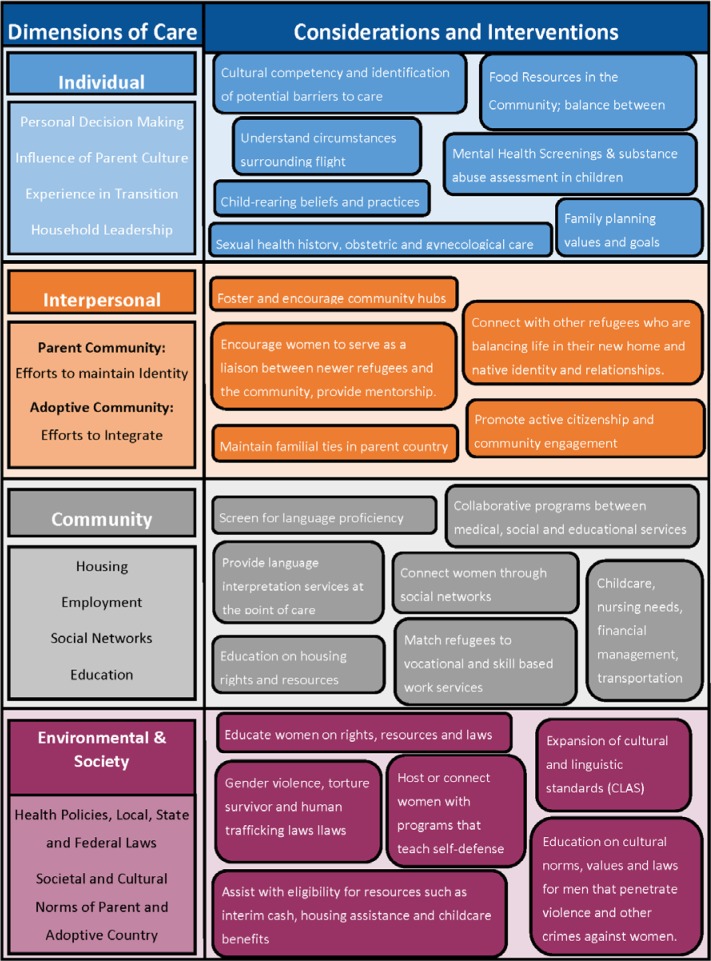
Summary of Recommendations in an Ecological Model.

A summary of the above recommendations in the ecological model is provided in Figure 3.

## 4. Bright Spots in Ecological Approaches to Refugee Care – S.M.AR.T Strategies in Primary Care

**Figure 4.**
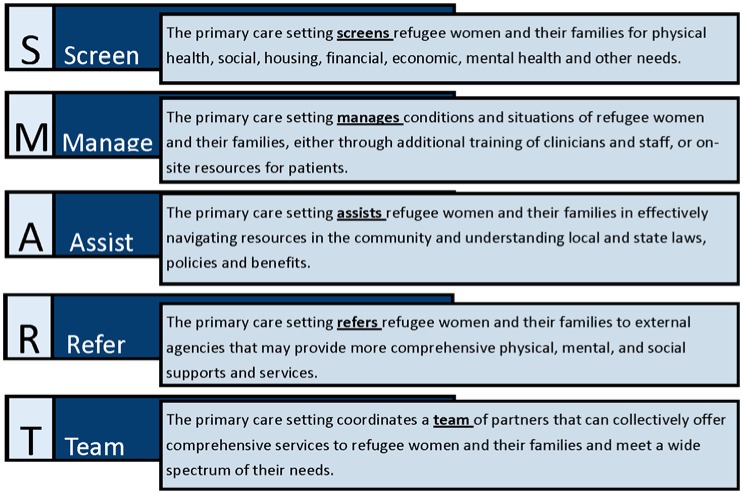
S.M.A.R.T. Strategies for Primary Care Settings in Refugee Women's Care. Source: Developed by authors, Drs. Olayiwola and Raffoul.

Undertaking a comprehensive, culturally specific effort to refugee integration involves multiple stakeholders, and can be overwhelming to conceptualize or implement. Using the healthcare system, and particularly the primary care system, as a portal of entry into services for refugee women may be a powerful strategy to ensure that women and their families can transition to their new societies in a wholesome manner. However, what is the specific role of the primary healthcare system? Although the evidence on the impact of social and environmental issues on healthcare is well described, there is still a challenge of directly addressing social needs of patients in clinical settings. This is due to a combination of factors, including disagreement on ownership of these issues, inadequate time, tools, resources and organizational capacity to address these factors, and insufficient economic incentives [40],[41]. Primary care settings may not be able to address all of the needs of refugee women, but can apply any or all of a framework we have developed, a “S.M.A.R.T” strategy ensure that the needs of their refugee women are met (Figure 4). S.M.A.R.T strategies include Screening, Managing, Assisting, Referring and/or Team Coordination for clients, and primary care settings may choose to apply different combinations based on resources, client needs, and community partnerships. As the value of such efforts is documented, economic value and policy support will hopefully follow, and in the future, demonstrate not only social justice benefits, but good business and organizational sense [42].

Through referrals by a few experts in the field of refugee health and our own networks, we identified a number of distinct programs that have exemplified both the ecological model and S.M.A.R.T strategy in their services. The authors conducted interviews and observations of key leadership in three of these programs that were accessible to us, representing both social service and health delivery settings, looking at both the ecology of their approach and how S.M.A.R.T approaches had been included in holistic refugee health and integration. These three international “bright spots” in such S.M.A.R.T strategies are described below through program descriptions and available published or public information, and not as a qualitative research study. These programs/models suggest that saving women refugees and their families through an ecological approach to primary care is indeed possible.

### 4.1. Public Social Welfare Centres — Belgian Federal Public Planning Service for Social Integration

In Belgium, community oriented primary care efforts have led to a continuous process of assessment and intervention through the work and activities of public care social welfare centres (PSWCs). PSWCs are autonomous legal institutions in every municipality, which are governed by an elected council and share a mission of guaranteeing every person a life in dignity, including a minimum income. The PSWCs are supported by the Belgian Federal Public Planning Service for Social Integration (PPS SI, website — www.mi-is.be) [43]. One of the missions of these Centres is to provide newcomers to Belgium, including immigrants, refugees and asylees, with comprehensive primary care as well as coordination of social services, orientation to national programming and laws, and health promotion. The University Center for Primary Care “Nieuw Gent” is an integrated welfare building. As part of the welfare services, this building also includes social services, a social restaurant, intercultural network, well baby clinic, and multiple neighborhood organizations. All interventions begin with community-based focus group discussions centered on perceived community health needs. Then, needs specific interventions and metrics are developed. Resulting interventions are often multi-tiered and engage various stakeholders, and empower communities in reaching their own health goals. This is a hybrid of S.M.A.R.T approaches, in which primary care and social care entities share responsibility for the health, wellness and integration of their most vulnerable. Cross training providers and staff in different agencies for intercultural S.M.A.R.T strategies has been critical to its success. The PSWCs and their partners have been central to the resettlement of Syrian and Congolese refugees in particular and the ecology of this approach traverses all components of our ecological model [44].

### 4.2. Newcomers Health Program — San Francisco Health Network

The San Francisco Health Network/San Francisco Department of Public Health has successfully run the Newcomers Health Program (NHP), in collaboration with the Family Health Center's Refugee Medical Clinic, for nearly 40 years. This program provides services to United States designated refugees, asylees, victims of trafficking and their family members including: initial health assessments and access to primary care services, health insurance coverage and transition, cross cultural training for resident physicians, nursing students and staff, culturally competent health workers, mental health assessment, collaboration and referrals to the Trauma Recovery Center/Survivors International, community resources and bi-directional linkages, medical interpretation, language-specific social support groups, joint training opportunities for refugees and asylees (including health worker training programs), education programs and more. Arabic, Burmese, Cantonese, Mandarin, Mongolian, Spanish, and Russian speaking staff offer services to hundreds of refugees every year, and the collaboration has served over 50,000 refugees and asylees since the 1970s [45],[46].

A feature story in *SF Gate* [46] in 2011 stated the following about the NHP team: “They explain a brand new medical system in which preventative treatments such as vaccinations and physicals are emphasized, and they connect patients with classes in English as a second language, job training programs, housing assistance, help getting a green card and assistance enrolling their children in the public schools. They've even been known to give demonstrations on how to ride the sometimes perplexing Muni system.” The Newcomers Health Program has also successfully addressed refugee integration through all components of our ecological model, using S.M.A.R.T approaches and an additional layer of workforce development.

### 4.3. Asian Health Services, a Federally Qualified Health Center — Oakland, California

The Asian Health Services' (AHS) Frank Kiang Medical Center took a proactive approach to including and incorporating care for the Burmese and Karen populations, two new emerging Asian refugee communities in Alameda County, California. Their strategy provides an important lens to how a primary care setting can approach planning, implementation and spread of S.M.A.R.T strategy for refugees. The opening of a new medical center in 2010 created capacity for AHS to accept new patients. The expanded capacity then provided an opportunity to actively plan and build in systems of care for these two emerging refugee groups [47]. A four-phase approach to operationalizing care for new populations with distinct languages, cultures, and migration histories helped to organize the different staffing roles/responsibilities and program structure. The first phase entailed outreach to the community and data analysis of the populations. The second phase consisted of internal infrastructure development — devising staffing roles, training, and hiring practices. The third phase is the development of clinical operations, particularly specific to the needs of refugee communities, such as providing enabling services like immigration services, health education, eligibility services, patient navigation and health navigation. The final phase entails evaluation of programs, quality improvement, sustainability planning and spread [48].

The S.M.A.R.T framework and multi-dimensional ecological strategy are modeled within the programming for the Burmese and Karen refugee patients, particularly in perinatal women and pediatric programs. The Frank Kiang Medical Center developed an “Empowering Mothers” and “Blossoming Babies” group visit program with individual breakout clinical sessions. The group visit model provides extended health education sessions, integrates behavioral health and oral health care in a trauma-informed way [49], assists with referrals, and at the same time, increases a sense of community and social cohesion amongst the participants in a linguistic and culturally concordant way [48].

Bachrach and colleagues also document a number of other successful models of medical and social integration, not particularly for refugee related services, in an important 2014 Commonwealth Fund publication [41].

## 5. Conclusion

The goal of integrating and incorporating refugee women and their families presents an opportunity for accepting communities to come together through stakeholder engagement, evaluate their current capacities and assess future needs. Considering the health and well-being of refugee women offers an important window to the ultimate integration and success of refugee families. Similarly, the primary care system of many receiving countries can serve as an integral portal to the larger society, through S.M.A.R.T strategies and collaboration.

We have outlined an ecological model and strategy that communities may use to embrace their fellow humans as refugees, formulate plans of action and partnerships, absorb and adopt an incoming population, and convert the shock and anguish that often accompanies refugee resettlement into positive energy. This was not a research study or outcomes-based in focus, but it will be important that programs, such as those we have highlighted here, collect relevant data on impact and outcomes, and build the evidence-base for their successes. This will improve their ability to scale, spread and replicate their models more broadly.

Failure to integrate refugee populations into dominant society will, among other things, contribute to the health disparities observed seen in racial and ethnic minorities, a gap that is yet to be closed even in periods of normalcy. An inadequately treated refugee crisis will likely only widen it [50]. Optimizing the health of women refugees through an ecological lens, using S.M.A.R.T primary care strategy, provides a unique and perhaps bold attempt to saving, building and strengthening refugee families across the world, through the ever so important pillars in families…women.

*“Refugees have been deprived of their homes, but they must not be deprived of their futures.”* UN Secretary-General Ban Ki-moon.

“*I can be changed by what happens to me, but I refuse to be reduced by it.”* Maya Angelou.
